# Same-day discharge (SDD) vs standard enhanced recovery after surgery (ERAS) protocols for major colorectal surgery: a systematic review

**DOI:** 10.1007/s00384-023-04408-7

**Published:** 2023-05-01

**Authors:** V. Zheng, I. J. Y. Wee, H. R. Abdullah, S. Tan, E. K. W. Tan, I. Seow-En

**Affiliations:** 1 Yong Loo Lin School of Medicine, National University of Singapore, Singapore City, Singapore; 2 Department of Colorectal Surgery, Singapore General Hospital, Singapore City, Singapore; 3 Department of Anaesthesiology, Singapore General Hospital, Singapore City, Singapore

**Keywords:** Colorectal surgery, Same-day discharge, Enhanced recovery after surgery, ERAS, SDD

## Abstract

**Background:**

Enhanced recovery after surgery (ERAS) programs are well-established, resulting in improved outcomes and shorter length of hospital stay (LOS). Same-day discharge (SDD), or “hyper-ERAS”, is a natural progression of ERAS. This systematic review aims to compare the safety and efficacy of SDD against conventional ERAS in colorectal surgery.

**Methods:**

The protocol was prospectively registered in PROSPERO (394793). A systematic search was performed in major databases to identify relevant articles, and a narrative systematic review was performed. Primary outcomes were readmission rates and length of hospital stay (LOS). Secondary outcomes were operative time and blood loss, postoperative pain, morbidity, nausea or vomiting, and patient satisfaction. Risks of bias was assessed using the ROBINS-I tool.

**Results:**

Thirteen studies were included, with five single-arm and eight comparative studies, of which one was a randomised controlled trial. This comprised a total of 38,854 patients (SDD: 1622; ERAS: 37,232). Of the 1622 patients on the SDD pathway, 1590 patients (98%) were successfully discharged within 24 h of surgery. While most studies had an overall low risk of bias, there was considerable variability in inclusion criteria, types of surgery or anaesthesia, and discharge criteria. SDD resulted in a significantly reduced postoperative LOS, without increasing risk of 30-day readmission. Intraoperative blood loss and postoperative morbidity rates were comparable between both groups. Operative duration was shorter in the SDD group. Patient-reported satisfaction was high in the SDD cohort.

**Conclusion:**

SDD protocols appear to be safe and feasible in selected patients undergoing major colorectal operations. Randomised controlled trials are necessary to further substantiate these findings.

**Supplementary Information:**

The online version contains supplementary material available at 10.1007/s00384-023-04408-7.

## Introduction

Enhanced recovery after surgery (ERAS) protocols are a set of evidence-based practices spanning the perioperative period, designed to optimise postoperative outcomes by minimising the physiological stress response and maintaining or rapidly restoring baseline function [1]. The concept that a structured, multimodal, multidisciplinary pathway can result in accelerated recovery after surgery first emerged in the early 2000s amongst patients undergoing colorectal surgery [2–4]. ERAS programmes are now well-established in colorectal surgery, with the benefits over traditional care demonstrated by several meta-analyses of randomised controlled trials [5–8].

In 2018, the international ERAS Society published their fourth set of consensus guidelines concerning perioperative care in elective colorectal surgery [9]. In December 2022, the American Society of Colon and Rectal Surgeons (ASCRS) and the Society of American Gastrointestinal and Endoscopic Surgeons (SAGES) have also collaborated to report updated clinical practice guidelines [10], which aim to lead international efforts in defining the optimal perioperative approach to colorectal surgery based upon the principles of ERAS.

Continued interest and innovation in the various aspects of ERAS have led to its application, in whole or in part, amongst unconventional patient cohorts. Thus far, the feasibility of ERAS pathways has been demonstrated for emergency/non-elective resections [11, 12], elderly patients [13, 14], low rectal cancer [15], inflammatory bowel disease [16], as well as cytoreductive surgery with hyperthermic intraperitoneal chemotherapy [17]. Regardless of patient profile, ERAS protocol compliance has been well documented as the most essential factor contributing to better postoperative outcomes [18–20]. Moreover, addressing clinicians’ resistance to change and rectifying long-standing but non-evidence-based practices are important to ensure successful implementation of ERAS programmes [20].

One of the most striking consequences of improved patient recovery with ERAS is a substantially reduced length of hospital stay (LOS). Various meta-analyses have consistently demonstrated that ERAS decreases the postoperative LOS by an average of two days, as compared to standard perioperative care [5, 6, 21]. There is increasing recognition that same-day discharge (SDD), defined as hospital discharge within 24 h of surgery, or "hyper-ERAS”, may be feasible for a select group of physiologically fit patients.

In 2015, Gignoux et al. reported the first five cases of outpatient colectomy with a postoperative stay of less than 12 h [22]. Thereafter, six prospective and seven retrospective cohort studies have evaluated SDD following colorectal surgery [23–35]. These studies consistently demonstrate the safety of SDD, without an increase in readmission or complication rates [24, 27, 28, 30]. Enhanced post-discharge patient assessment was often performed, through teleconferencing, mobile applications, or telephone interviews.

Given the growing adoption of SDD as a natural progression of ERAS with an increasing amount of contemporaneous evidence, a systematic review is timely to determine the overall feasibility of “hyper-ERAS” for colorectal surgery and discuss the applications of individual components of SDD.

## Methods

### Search process

This study protocol was prospectively registered in PROSPERO (394793). This study was performed according to the Cochrane Handbook of Systematic Reviews and Meta-analysis version 6.2 (2021), and the Preferred Reporting Items for Systematic Reviews and Meta-Analyses (PRISMA 2020) statement guidelines [36]. In consult with our institution’s librarian, we derived an exhaustive permutation of the following Medical Subject Headings (MeSH) (expanded) terms: “colon surgery”, “colorectal surgery”, “rectal surgery”, “colectomy”, “colon resection”, “ERAS”, “enhanced recovery” “23 h”, “same day discharge”. Using this search strategy, an electronic search was performed on 25 December 2022 in the following databases: Medline (via PubMed), EMBASE, Cochrane databases, and the ClinicalTrials.gov website, to identify all published and indexed studies investigating same-day discharge protocols after colorectal surgery. A manual search of the reference lists of relevant studies was conducted to identify additional studies for potential inclusion. In addition, a search of grey literature (conference proceedings, theses, published abstracts) was performed, as per recommendation in the AMSTAR 2 checklist [37].

### Inclusion and exclusion criteria

Both randomised and non-randomised controlled trials were included if outcomes were reported for patients undergoing same-day/23-h discharge protocols after colorectal resection surgeries. Given the relative lack of comparative studies, single-arm studies with reported outcomes and extractable data were included. Non-English studies as well as English studies with no extractable data were excluded as there were no available translators at our institution.

### Selection of studies

**Fig. 1:**
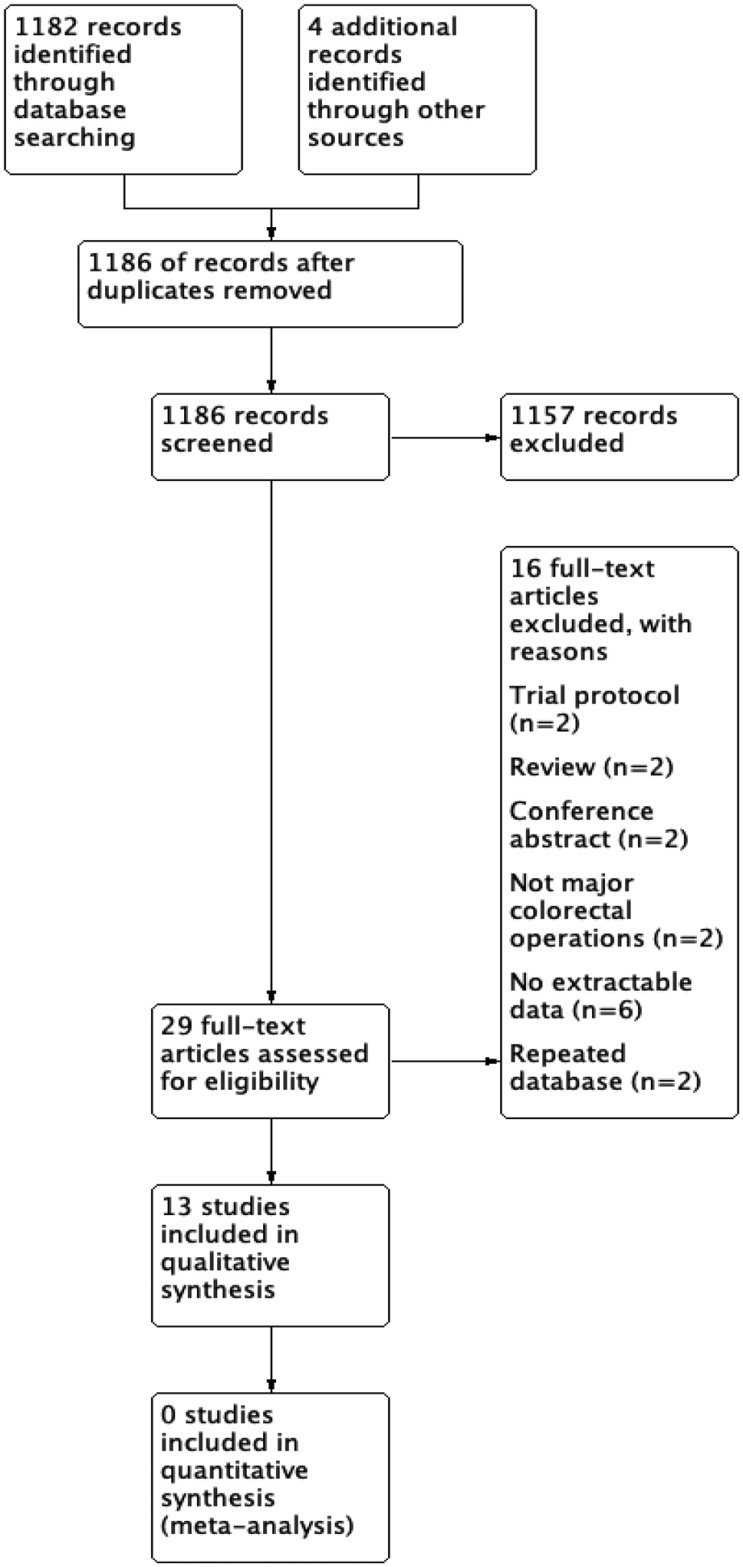
Prisma flow diagram for the systematic review showing database searches, number of abstracts screened, and full texts retrieved

As seen in the PRISMA diagram (Fig. 1), the selection of studies was conducted in two distinct stages. Firstly, two reviewers (VZ, IW) independently screened the studies for preliminary inclusion by their titles and abstracts, and the full text of these studies would then be re-assessed for final inclusion. The senior author would be the arbiter to resolve differences of opinions regarding the studies’ eligibility by consensus.

### Outcomes of interest and data extraction

Outcomes of interest included intraoperative and postoperative variables which were extracted independently by two authors using a standardised proforma. Primary outcomes were readmission rates and length of hospital stay. Secondary outcomes were operative time, intraoperative blood loss, conversion to open surgery, postoperative morbidity, pain, nausea and vomiting, patient satisfaction, and protocol compliance. In addition to the outcomes above, we extracted the following data from each study: first author, year, type of publication, age, gender, perioperative protocols, type of surgeries, stage of tumour (when applicable), American Society of Anaesthesiologists (ASA) scores, comorbidities. Data extraction was performed in an independent fashion by VZ and IW, and conflicts were resolved either by consensus or with discussion with the senior author.

### Statistical analysis

Where possible, statistical analyses were performed using the RevMan 5.4 software (The Nordic Cochrane Centre, Copenhagen, Denmark), where pooling of risk ratio (RR) was performed for dichotomous variables; and weighted mean difference (WMD) or standardised mean difference (SMD) conducted as the summary statistic for continuous variables. Statistical heterogeneity was assessed using the I^2^ statistic. A random-effects model was chosen when the I^2^ statistic was greater than 50%, and a fixed-effects model otherwise. Results were reported with 95 percent confidence intervals (95% CI), and a P value of less than 0.05 was treated as statistically significant.

Pair-wise meta-analyses were not performed given small number of comparative studies, as well as the significant heterogeneity in study protocols and reporting of results. Subgroup analyses were also not performed given the lack of data stratification. Thus, a narrative systematic review was undertaken.

### Assessment of bias

The Risk of Bias in Non-randomised Studies – of Interventions (ROBINS-I) tool [39] was utilised to assess the quality and risks of bias from confounding, selection of participants, classification of interventions, deviations from intended intervention, missing data, measurement of outcomes, and selection of reported results. Publication bias was not assessed using the funnel plot and Egger’s regression test since there were less than 10 studies.

## Results

### Systematic search

Out of a total of 1,185 papers, 29 were included after title and abstract review. These 29 papers were reviewed in entirety and 13 were included in the final systematic review, of which five were single-arm and eight were comparative studies (one randomised controlled trial). The systematic search process and reasons for exclusion are stated in Fig. 1.

The 13 studies comprised a total of 38,854 patients, of whom 1622 received the SDD protocol and 37,232 received conventional ERAS protocol. Of the 1622 patients receiving SDD protocol, 1590 patients (98%) were successfully discharged within 24 h. Gignoux et al. (2015) contributed the least number of patients to the SDD pool (0.3%), while McKenna et al. contributed the most (55.9%). Across the studies, the mean age ranged from 57.4 to 67.0 years in the SDD arm, and 56.5 to 72.6 years in the conventional ERAS arm. The proportion of males ranged from 35 to 80% in the SDD arm, and 40.3% to 62.5% in the conventional ERAS arm.

All procedures were minimally invasive surgeries (MIS), done via laparoscopic or robotic methods. Surgeries performed included colectomies, anterior resections, stoma closures, Hartmann’s reversals and parastomal hernia repairs. The indications for surgery included both benign and malignant conditions. All except one single-arm study [34] reported the indications for surgery. The proportion of patients with malignant indications ranged from 20 to 100% in the SDD arm, and 42% to 100% in the conventional ERAS arm. Patients in most studies were discharged home. Four studies [25, 29, 31, 33] only included patients in the SDD arm if they stayed close to the hospital (less than 30 min drive away). Detailed baseline study characteristics are shown in Table 1. The ERAS and follow-up protocols are reported in Table 2.

**Table 1:** Baseline study characteristics of studies included in the systematic review of SDD vs standard ERAS following major colorectal surgery

	Variables								
First author, Year	Type of study	No. of patients	Male gender	Mean age, years	ASA score	Type of surgery (MIS/ open)	Procedure performed	Indication for Surgery	Stage of cancer
Bednarski et al. (2019) [23]	RCT	SDD: 14 Control: 16	SDD: 6 (42.9%) Control: 10 (62.5%)	SDD: 58.7 (sd 12.6) Control: 59.3 (sd 10.2)	SDD: ASA 3: 14 Control: ASA 2: 2 ASA 3: 14	MIS	SDD: - Right colectomy: 8 (57.2%) - Left colectomy: 3 (21.4%) - Anterior resection: 3 (21.4%) Control: - Right colectomy: 8 (50.0%) - Left colectomy: 1 (6.2%) - Anterior resection: 7 (43.8%)	All malignant	SDD: AJCC I: 6 (42.8%) AJCC II: 4 (28.6%) AJCC III: 2 (14.3%) AJCC IV: 1 (7.1%) unresectable polyp: 1 (7.1%) Control: AJCC I: 5 (31.3%) AJCC II: 6 (37.5%) AJCC III: 4 (25.0%) unresectable polyp: 1 (6.2%)
Brandt et al. (2013) [24]	Retrospective	SDD: 24 Control: 209	SDD: 12 (50.0%) Control: 106 (51.0%)	SDD: 64 (range 35–81) Control: 70 (range 16–90) P = 0.018	SDD: ASA < 3: 22 (92.0%) ASA ≥ 3: 2 (8.0%) Control: ASA < 3: 175 (84.0%) ASA ≥ 3: 34 (16.0%) P = 0.916	MIS	SDD: - Sigmoid resection: 16 (66.7%) - Right hemicolectomy: 8 (33.3%) Control: - Sigmoid resection: 106 (50.7%) - Right hemicolectomy: 103 (49.3%)	All malignant	Not stated
de Azevedo et al. (2021) [27]	Retrospective	SDD: 237 Control: 427	SDD: 83 (35.0%) Control 172 (40.3%) P = 0.182	SDD: 60 (47.0–68.5) Control: 61 (47.0–72.0) P = 0.083	SDD: ASA 1: 122 (51.5%) ASA 2: 115 (48.5%)	MIS	SDD: - Right colectomy: 50 (21.2%) - Left colectomy: 7 (3.0%) - Partial colectomy: 13 (5.5%) - Proctocolectomy: 8 (2.0%) - Rectosigmoidectomy: 152 (64.1%) -Abdominoperineal amputation: 2 (0.8%) - Total colectomy: 2 (0.8%) - Other colorectal procedures 10 (4.2%) Control: - Right colectomy: 80 (18.7%) - Left colectomy: 19 (4.4%) - Sigmoidectomy: 7 (1.6%) - Partial colectomy: 15 (3.5%) - Proctocolectomy: 0 (0.0%) - Rectosigmoidectomy: 224 (52.5%) - Abdominoperineal amputation: 16 (3.7%) - Total colectomy: 23 (5.4%) - Other colorectal procedures 36 (8.3%)	SDD: - Malignant: 113 (47.7%) - Benign: 124 (52.3%) Control: - Malignant: 301 (70.7%) - Benign: 125 (29.3%) P < 0.001	AJCC I: 48 (20.3%) AJCC II: 29 (12.2%) AJCC III: 22 (9.3%) AJCC IV: 7 (3.0%) P = 0.166
Lee et al. (2022) [29]	Prospective	SDD: 48 recruited for SDD *of which 37 were discharged on day of surgery without subsequent ED visit in first 72 h* *(7 failed SDD criteria and required admission, 4 experienced issues/complications)* Control: 73	SDD (out of the 48 patients): 22 (46.0%) Control: 43 (59.0%) P = 0.158	SDD (out of the 48 patients): 60.2 (sd 10.5) Control: 56.5 (sd 13.1) P = 0.111	SDD (out of the 48 patients): ASA 1: 4 (8.0%) ASA 2: 27 (55.0%) ASA 3 + : 17 (35.0%) Control: ASA 1: 4 (5.0%) ASA 2: 38 (52.0%) ASA 3 + : 31 (43.0%) P = 0.499	MIS	SDD: - Right colectomy: 14 (29.0%) - Left or sigmoid colectomy: 12 (25.0%) - Anterior resection: 7 (15.0%) - Stoma closure: 15 (31.0%) Control: - Right colectomy: 33 (45.0%) - Left or sigmoid colectomy: 22 (30.0%) - Anterior resection: 11 (15.0%) - Stoma closure: 7 (10.0%) P = 0.022	SDD: - Malignant: 25 (52.0%) - Benign: 23 (48.0%) Control: - Neoplasm: 47 (64.0%) - Benign: 26 (36.0%) P = 0.027	Not stated
McKenna et al. (2020) [30]	Retrospective	Discharged POD 0–1: 906 Discharged POD 2: 6825 Discharged POD 3–5: 28,795	Discharged POD 0–1: 437 (48.0%) Discharged POD 2: 3411 (50.0%) Discharged POD 3–5: 13,586 (47.0%) male P = 0.0002	*Median* Discharged POD 0–1: 60 (IQR 52–68) Discharged POD 2: 60 (IQR 51–68) Discharged POD 3–5: 61 (IQR 51–70) P < 0.0001	Discharged POD 0–1: ASA 1: 47 (5.0%) ASA 2: 560 (62.0%) ASA 3: 291 (32.0%) ASA 4: 8 (1.0%) Discharged POD 2: ASA 1: 263 (4.0%) ASA 2: 4273 (63.0%) ASA 3: 2286 (34.0%) Discharged POD 3–5: ASA 1: 955 (3.0%) ASA 2: 16,383 (57.0%) ASA 3: 11,419 (40.0%) P < 0.0001	MIS	Discharged POD 0–1: - Segmental colectomy: 504 (56.0%) - Colectomy with removal of terminal ileum and ileocolostomy: 260 (29.0%) - Segmental colectomy with coloproctostomy: 142 (16.0%) Discharged POD 2: - Segmental colectomy: 2911 (43.0%) - Colectomy with removal of terminal ileum and i-leocolostomy: 1783 (26.0%) - Segmental colectomy with coloproctostomy: 2131 (31.0%) Discharged POD 3–5: - Segmental colectomy: 13,070 (45.0%) - Colectomy with removal of terminal ileum and ileocolostomy: 6956 (24.0%) - segmental colectomy with coloproctostomy: 8769 (31.0%) P < 0.0001	Discharged POD 0–1: - Malignant: 236 (26.0%) - Benign: 670 (74.0%) Discharged POD 2: - Malignant: 2861 (42.0%) - Benign: 3964 (58.0%) Discharged POD 3–5: - Malignant: 12,622 (44.0%) - Benign: 16,173 (56.0%) P < 0.0001	Not stated
Popeskou et al. (2022) [31]	Retrospective	SDD: 51 Control: 782	SDD: 66.0% Control: 47.0%	*Median* SDD: 67 (range 59–72) Control: 70 (range 60–78)	SDD: ASA 1: 10 (19.6%) ASA 2: 35 (68.6%) ASA 3: 6 (11.8%) Control: ASA 1: 110 (14.4%) ASA 2: 482 (62.9%) ASA 3: 168 (21.9%) ASA 4: 6 (0.8%)	MIS	SDD: - Ileocolonic resection: 2.0% - Right hemicolectomy: 57.0% - Extended right hemicolectomy: 0.0% - Left hemicolectomy: 2.0% - Sigmoidectomy: 2.0% - High anterior resection: 37.0% Control: - Ileocolonic resection: 2.0% - Right hemicolectomy: 42.0% - Extended right hemicolectomy: 8.0% - Left hemicolectomy: 4.0% - Sigmoidectomy: 7.0% - High anterior resection: 36.00%	SDD: - Malignant: 45 (88.2%) - Benign: 6 (11.8%) Control: - Malignant: 590 (75.4%) - Benign: 192 (24.6%)	Not stated
Levy et al. (2009) [33]	Prospective	SDD: 10 Control: 30	SDD: 4 (40.0%) Control: 17 (56.7%) P = 0.46	SDD: 60 (range 43–72) Control: 69 (range 33–91) P = 0.04	SDD: ASA 1: 1 (10.0%) ASA 2: 9 (90.0%) Control: ASA 1: 1 (3.3%) ASA 2: 24 (80.0%) ASA 3: 5 (16.7%) P = 0.44 for ASA 1 P = 0.66 for ASA 2 P = 0.31 for ASA 3	MIS	SDD: Right hemicolectomy: 3 (30.0%) Left hemicolectomy: 1 (10.0%) Sigmoid colectomy: 2 (20.0%) High anterior resection: 2 (20.0%) Total mesorectal excision with no ileostomy: 2 (20.0%) Control: Not stated	SDD: - Malignant: 9 (90.0%) - Benign: 1 (10.0%)	Not stated
Tweed et al. (2022) [32]	Prospective SDD cohort vs Retrospective control cohort	SDD: 41 (of which 33 Patients were discharged within 23 h) Control: 75	SDD (out of 41): 27 (65.9%) Control: 33 (44%) P = 0.024	*Median* SDD (out of 41): 67 (IQR 57–73) Control: 72.6 (IQR 63.6–75.7) P = 0.007	SDD (out of 41): P = 0.156 ASA 1: 1 (2.4%) ASA 2: 40 (97.6%) Control: ASA 1: 8 (10.7%) ASA 2: 67 (89.3%)	MIS	SDD (out of 41): - Left hemicolectomy: 3 (7.3%) - Right hemicolectomy: 19 (46.3%) - High anterior resection: 18 (43.9%) - Transverse colectomy: 1 (2.4%) Control: - Left hemicolectomy: 6 (8.0%) - Right hemicolectomy: 28 (37.3%) - High anterior resection: 37 (49.3%) - Transverse colectomy: 1 (1.3%) - Total colectomy: 1 (1.3%) - Subtotal colectomy: 2 (2.6%)	All malignant	Not stated
Campbell et al. (2022) [25]	Retrospective	7	4 (57.1%)	57.4	Not stated	MIS	2 Right colectomy (28.6%) 4 Anterior resection (57.1%) 1 Hartmann’s reversal (14.3%)	malignant: 6 (85.7%) Benign: 1 (14.3%)	Not stated
Curfman et al. (2022) [26]	Retrospective	115	48 (41.7%)	58.9	Not stated	MIS	Anterior resection: 61 (53%) Right colectomy: 25 (21.7%) Caecectomy: 9 (7.8%) Sigmoidectomy: 6 (5.2%) Parastomal hernia: 5 (4.3%) Transverse colectomy: 3 (2.6%) Proctectomy: 3 (2.6%) Left colectomy: 3 (2.6%)	Not stated	Not stated
Dobradin et al. (2013) [28]	Retrospective	7	3 (42.9%)	63.1	ASA 1: 1 ASA 2: 4 ASA 3: 2	MIS	Right hemicolectomy: 4 (57.1%) Sigmoid colectomy: 3 (42.9%)	Malignant: 4 (57.1%) Benign: 3 (42.9%)	1 patient with T2N1 1 patient with T3N1 1 patient with T2N0 1 patient with T1N0
Gignoux et al. (2015) [22]	Prospective	5	4 (80%) (discharge < 12 h after admission)	64.0	ASA 1: 1 (20.0%) ASA 2: 4 (80.0%)	MIS	Type of colectomy not specified (all resections involved lesions at the sigmoid colon)	Malignant: 1 (20.0%) Benign: 4 (80.0%)	Not stated
Gignoux et al. (2019) [35]	Prospective	SDD: 157 of which only 144 were ambulatory, 11 required admission: - 3 patients with social problems - 4 with medical problems - 4 with operative difficulties	SDD (out of 157 patients): 87 males (55.4%)	SDD (out of 157 patients): mean age: 59.1 (sd 11.4)	Not stated	MIS	Left colectomy: 116 (73.8%) Right colectomy: 18 (11.4%) Rectosigmoid resection with intraperitoneal colorectal anastomosis: 17 (10.3%) Splenic flexure colectomy: 3 (1.9%) Total colectomy 1 (0.6%)	Malignant: 54 (34.3%) Benign: 103 (65.7%)	Not stated

Reported as n/%

*SDD* same-day discharge, *ERAS* enhanced recovery after surgery, *RCT* randomised controlled trial, *ASA* American Society of Anaesthesiology, *MIS* minimally invasive surgery, *AJCC* American Joint Committee on Cancer, *POD* post-operative day, *DVT* deep vein thrombosis, *GA* general anaesthesia, *SBIO* small bowel intestinal obstruction, *SD* standard deviation, *IQR* interquartile range

**Table 2:** ERAS and follow-up protocols of studies included in the systematic review of SDD vs standard ERAS following major colorectal surgery

First author, Year	ERAS protocol	Follow-up protocol	Patient selection for SDD and discharge criteria (if stated)	Discharge destination
Bednarski et al. (2019) [23]	Pre-operative: - Education - Bowel preparation (mechanical + antibiotics) - Pre-medications (Celecoxib, Pregabalin, Tramadol) Intra-operative: - Medications (Dexamethasone, Tylenol, Dexmedetomidine, Lidocaine, Ondansetron, Opioid sparing analgesic strategies - Goal directed fluid therapy (Using non-invasive cardiac output monitor) Post-operative: - POD 0: Optimised fluids, managed out of bed to chair/ambulating - POD 1: Liquid diet + Toast/crackers, advance to solid as tolerated, out of bed to chair/ambulating	Tele Recovery appointment with the surgical team on POD 2. Instructions to patients on videoconferencing and instant messaging, and provision of an iPad for communication/video conferencing with the healthcare team. The trial coordinator monitored the instant messaging and communicated with the healthcare team. Outpatient intravenous fluid hydration at an ambulatory infusion centre on POD 2/POD 3 when the patient identified to have inadequate oral intake or thought to be at high risk of dehydration following video-conferencing.	Patient randomisation after surgery.	Discharged to own home or hotel lodging in the immediate surrounding area. Patients were required to reside within close travelling distance (2–3 h) of the hospital.
Brandt et al. (2013) [24]	Pre-operative: - Pre-operative bowel preparation with a rectal enema on the night and morning prior to surgery (only for sigmoidectomy patients) - On day of surgery, all patients received 200 ml Provide Xtra Intra-operative: - Induction of GA with propofol and fentanyl - Urinary catheters and oral tubes were only used during surgery. No drains were used Post-operative: - Postoperative pain control: oral Paracetamol, Ibuprofen, Morphine (if needed) - All patients mobilised on day of surgery and given general diet	Planned clinic review after POD 10.	Postoperative patient opt-in process for early discharge.	Discharged to own home.
de Azevedo et al. (2021) [27]	Pre-operative: - All patients had mechanical bowel prep - Normal meal the previous evening, liquid diet until 4 h before surgery - Prophylactic antibiotics - No pre-operative anaesthesia induction Intra-operative: - GA with epidural blockage and with intra-abdominal pressure of 12 mmHg - Maintained in hyperoxygenation state with limited IV fluid intake, minimised narcotic use - Hypothermia controlled with IV warm fluids, warming blankets and mattresses - Local anaesthesia at incision site or transversus abdominis plane block performed in all patients - No gastric tube, urinary catheter or abdominal drains were left after surgery Post-operative: - Prokinetics (bromopride 3 × a day) - Regular analgesics (Dipyrone and tramadol) - Tolerate liquid diet when awake and soft diet by end of the day - Early ambulation recommended After discharge: - Oral analgesia given, DVT prophylaxis done with daily ambulation at home - For 7 days, patients recommended to remain at locations with easy access to hospital	Scheduled phone call on POD3. Patients were able to contact the healthcare team via direct telephone calls or messages. In-person consultation and physical examination on POD 7, 21 and 45. In the presence of significant changes in pulse, blood pressure, temperature and complaints of an increase in pain, abdominal distention and non-passage of flatus or faeces, patients referred for full clinical examination and additional testing/studies as needed by a staff surgeon	Preoperative selection based on clinical and social factors.	Discharged to own home.
Lee et al. (2022) [29]	- Anastomoses performed intracorporeally - Pfannenstiel incision for specimen extraction - Bilateral transversus abdominis block at the end of procedure - Patients kept in PACU until ready for discharge - Clear fluid tray and 1 nutritional drink (boost or ensure) provided - Patient encouraged to mobilise in PACU - Assessed 4-6 h after arrival in PACU and directly discharged if they tolerated liquid diet without nausea or vomiting, had adequate pain control with oral analgesics, were ambulating and urinating independently, and had no evidence of complications.	Mobile application for direct communication with surgeons. Daily health check questionnaires were administered upon discharge and continued to POD 7, focusing on GI recovery, fever, and pain.	Preoperative selection based on clinical and social factors.	SDD offered only if patient lived within proximity of the hospital (50 km or 30 min drive).
McKenna et al. (2020) [30]	Specific ERAS pathways used were unknown	Specific follow-up protocol was unknown.	Retrospective study without elaboration on selection criteria.	Discharged to own home
Popeskou et al. (2022) [31]	Pre-operative: - Anaesthetic/Cardio-Pulmonary Evaluation - Nutritional and General status optimisation - Drug charts filled/Discharge plans initiated - Patient Education on ERP - Stoma discussion - 2 × CLINUTREN^®^PRELOAD™(powdered neutral-tasting carbohydrate loading drink mix) the night before - 1 × CLINUTREN^®^PRELOAD™(powdered neutral-tasting carbohydrate loading drink mix) 2 h before the procedure - Minimal to no bowel preparation - No premedication Intra-operative: - Minimal Invasive - Spinal Anaesthesia - Short-acting anaesthetic agents - Avoid NG tubes - Avoid fluid overload - Cefuroxime 1.5 g/metronidazole 500 mg at induction - Urinary catheters - Flowtron Anti-Embolic stockings - Warming - Infiltration of all stab wounds with local anaesthetic Post-operative: - Free Fluids - Simple Analgesia - Avoid Opioids - Early mobilisation - Stop IV fluids/Remove catheters - Low-fibre diet - Regular self-medication	Patients were able to contact the colorectal unit via direct telephone calls. Clinic review 1 month post-operatively.	Preoperative selection based on clinical and social factors.	SDD offered only if patient lived within proximity of the hospital (30 min drive)
Levy et al. (2009) [33]	Protocol used: 1. Preoperative education 2. Eat and drink normally the day before surgery 3. Avoidance of bowel preparation 4. Preoperative carbohydrate drink: 100 g of PreLoad^®^ in 800 ml of water the night before surgery and 50 g of PreLoad^®^ in 400 ml of water 2 to 3 h before surgery 5. Avoidance of preoperative sedatives 6. Spinal anaesthesia before skin incision 7. Target-driven, oesophageal Doppler–directed fluid therapy 8. Upper body air-heating cover 9. Avoidance of abdominal drains 10. Avoidance of nasogastric tube 11. Oral fluid in recovery or on arrival at ward 12. Sit out on afternoon or evening of surgery 13. Normal diet from dinner 14. Mobilization on evening of surgery, with walking on the spot for 3 to 5 min 15. Central venous saturation measurement on evening of Day 0 16. Termination of urinary drainage at midnight on Day 0 17. Termination of intravenous fluids at 18 to 20 h after surgery 18. Aperient given 19. Discharged home if tolerating breakfast, passing urine, and comfortable 20. Phone on evening of discharge with follow-up in clinic at end of that week Significant modifications: - Use of spinal analgesia using a local anaesthetic (2.5 ml heavy bupivacaine 0.5%) and 0.25 mg diamorphine rather than epidural analgesia. - Despite targeting intravascular volume using oesophageal Doppler during surgery, three of the ten patients required further colloid boluses to restore or normalize oxygen delivery on the evening of surgery.	On the evening of discharge, the research registrar called the patient to ensure that there were no problems. Patients were given a contact number that they could call if there was a problem. Clinic review POD 3.	Preoperative selection based on clinical and social factors.	SDD offered only if patient lived within proximity of the hospital (< 10 miles)
Tweed et al. (2022) [32]	Pre-operative: - Dedicated preoperative counselling - Baseline assessment with physical examination, electrocardiogram on indication and standard laboratory work-up - Nutritional screening by dietician - Fasting 6 h prior to surgery for solid food and 2 h prior to surgery for liquids - Oral carbohydrate loading for non-diabetic patients at least 2 h prior to surgery - Bowel preparation with bisacodyl (2 tablets of 5 mg the night before surgery and 1 tablet the morning of surgery) for patients scheduled for left-sided colon surgery - Preoperative analgesia with 1000 mg Paracetamol and 600 mg Gabapentin (300 mg if glomerular filtration rate < 60 ml/min or age > 70 years) Intraoperative: - Spinal anaesthesia (Prilocaine)* prior to induction of general anaesthesia - Use of short-acting total intravenous anaesthesia (propofol, remifentanil and ketamine) - Restrictive fluid management with continuous perfusion of Ringer Lactate 3 ml/kg/h - Deep neuromuscular blockade (Rocuronium bromide perfusion) - Lung protective ventilation (Total Volume 6–8 ml/kg; minimum FiO2 and optimal PEEP) - Adequate temperature regulation with forced air warming and core temperature monitoring - Starting intra-abdominal pressure at 12 mmHg with a gradual decrease to 8 mmHg - Minimally invasive surgery with intracorporal anastomosis - Extraction of specimen through a suprapubic Pfannenstiel incision, no additional mini-laparotomy performed Post-operative – directly: - Postoperative pain management consisted of Paracetamol (4 × 1000 mg), Meloxicam (1 × 7.5 mg daily for 3 days). If indicated, 5–20 mg Oxynorm (per os) or opioids in the form of Piritramide (intravenously) were given - Intake and gastro-intestinal motility were stimulated by offering an ice lolly on the recovery ward - Mobilisation was actively stimulated - If an urinary catheter was placed; this was removed before 10:00 PM on the surgical ward POD 1: - Routine physical examination by the ward physician - Evaluation of recovery and readiness for discharge - Expectation management regarding postoperative recovery and provision of an information booklet about postoperative recovery *Initially, patients received a combination of spinal anaesthesia with bupivacaine-glucose and morphine intrathecally prior to general anaesthesia. After the first 9 included patients, this was switched to intrathecal Prilocaine in combination with total intravenous anaesthesia (TIVA), because of an increase in the incidence of nausea and urinary retention postoperatively. The remaining included patients received spinal anaesthesia with 60–80 mg Prilocaine (weight based)	Telephonic aftercare was conducted by the nurse on the evening of discharge and on POD 4 to evaluate recovery and assess the presence of abnormal vital signs. Clinic review one week after discharge	Preoperative selection based on clinical and social factors.	Discharged to own home
Campbell et al. (2022) [25]	- Pre-operative ERAS education class for all patients - Alvimopam pre-operatively - Multimodal pain control algorithm including preop acetaminophen, gabapentin, NSAIDs and low dose narcotics - Ultrasound guided transversus abdominis plane block performed by anaesthesia in PACU - Liquid diet in recovery unit - Recommended to have full liquid diet in POD1 and advance to regular diet as tolerated - All patients ambulate independently before discharge - DVT prophylaxis given pre-operatively and in recovery unit, followed by injections at home - Foley catheters were removed postoperatively, and if the patient was unable to void, were replaced and the patient was discharged with a leg bag - Additional ostomy teaching was performed, intravenous fluids given - Patients were advised to stay close to the hospital postoperatively	Clinic review arranged within the first 4 postoperative days, and patients were contacted by telephone daily during that period. All patients had in-person 2-week clinic review.	Preoperative selection based on clinical and social factors.	Patients were advised to stay close to the hospital postoperatively Two of the seven patients chose to stay in a nearby hotel for 24 to 48 h as they lived more than 30 min away from the hospital
Curfman et al. (2022) [26]	Pre-operative: - 2 h-preoperative clear liquid diet, carbohydrate loading, mechanical bowel preparation, oral antibiotic bowel preparation, use of regular order set, prehabilitation (unchanged from ERAS) - Patient education, family/support system education, specific criteria for patient selection (advanced from ERAS) 1. Discuss with patients ileostomy teaching, importance of dehydration avoidance, expected milestones, and discharge criteria Intra-operative: - Surgical site infection bundle use, multimodal opioid sparing pain control, pre-op antiemetics guided by screening, multimodal nausea prophylaxis, tailor crystalloids to avoid excess, goal directed fluid therapy, avoidance of drains and tubes (unchanged from ERAS) - Minimally invasive approach and minimally invasive extraction (advanced from ERAS) 1. The use of minimally invasive surgery was employed in all procedures, with a majority performed robotically. Not described in ERAS, a minimally invasive manipulation approach was employed as well. It avoids regular tissue grabbing, and instead applies positional changes and sweeping methods for exposure and manipulation 2. Minimally invasive extraction method. When possible, natural orifice extraction or extraction via ostomy take down sites was employed. Post-operative: - Early mobilisation, immediate resumption of diet, early discontinuation of IV fluid, multimodal opioid sparing pain control, multimodal antinausea regimen, no urinary catheter use (unchanged from ERAS) - Post-operative education, post-operative patient physician communication (advanced from ERAS) 1. Patient preference of communication method (phone or Internet medical portal) is confirmed and recorded in the EMR at the preoperative visit. A provider routinely contacts the patient after discharge to assess their progress, address any questions, and analyse for any potential complications.	Patients were called by the operative physician on POD 1, and by a colorectal specific physician’s assistant on POD 3 to assess for any issues and answer questions. Clinic review between POD 5 and POD 7 and scheduled for further routine postoperative reviews from that point.	Preoperative selection based on clinical and social factors.	Discharged to own home
Dobradin et al. (2013) [28]	Preoperative: - Patient counselling: perioperative care, surgery, and LOS - Bowel preparation: 1 gallon of polyethylene glycol electrolyte solution 1 day before operation - IV antibiotics: 1 h before operation Intra-operative: - Laparoscopic surgery - No use of abdominal drains, nasogastric tubes, prokinetic agents, opioid antagonists - No use of epidural anaesthesia Postoperative: - Immediate postoperative liquid diet - Early ambulation initiated within 24 h - Prompt removal of bladder catheter - Pain management: 10 mg of Ketorolac by mouth - Follow-up at 1 week advised - Laxatives, opioid antagonists, and prokinetic agents were not used	Clinic review 1 week after discharge or earlier if necessary.	Retrospective study without elaboration on selection criteria.	Discharged to own home
Gignoux et al. (2015) [22]	Pre-operative: - Cancer immunonutrition was prescribed for 7 days - No bowel preparation - Fasting except for oral intake of 400 mL of sweetened clear liquids two hours before surgery - Pre-operative analgesia of Paracetamol Intra-operative: - Laparoscopic technique - Hyperoxygenation with FiO2 80% - Anaesthesia agents with short duration of action - Limited IV fluid during surgery - Intra-op and post-op analgesia aimed to minimise narcotic use by employing multimodal combination of Paracetamol, Nefopam, Lidocaine, Ketamine, Magnesium Sulphate, Tramadol and Morphine - Prevention of post-operative nausea vomiting by intra-operative administration of droperidol and dexamethasone Post-operative: - No NG tube, urinary catheter, or drains - No IV perfusion - Only oral analgesia – Paracetamol, Tramadol and Nefopam (per patient request for first 24 h) - Encouraged to walk and food allowed in semi-solid form 2 h after return to ward - Prevention of ileus and associated post-operative nausea vomiting by chewing gum and oral magnesium supplements 3 × daily - Thromboembolic prophylaxis – daily injection of enoxaparin SC for 10 days	Daily home visits every afternoon by a nurse for the first 10 postoperative days with clinical monitoring Daily morning phone calls	Pre-operative patient selection but discharge allowed based on Chung score [39] Discharge was authorized if the patient met the exit criteria according to the Chung score [39].	Discharged to own home
Gignoux et al. (2019) [35]	Pre-operative: - Immunonutrition (Oral Impact, 200 mL 3times per day for 7 days) was prescribed to patients with cancer - No mechanical bowel preparation - All eligible patients were asked to perform ‘‘modern fasting’’ which is a normal meal the previous evening, followed by oral intake of a 400 mL carbohydrate-loaded drink2 hours before surgery - Prophylactic antibiotics were administered intravenously at the induction of anaesthesia Intra-operative: - Infiltration of local anaesthetic (ropivacaine 7.5 mg, 30 mL) at the incisions site or transverse abdominal plane block - No preoperative sedative medication, hyperoxygenation (if O2at 80%), anaesthetic agents with a short duration of action, and limited intravenous fluid intake during surgery (lower total target of 5 mL/kg/h amounting to less than1000 mL) - Vessel sealing was performed using 38hermos-fusion scissors. - No gastric tube, urinary catheter, or abdominal drains were inserted. - Skin closure was performed with absorbable sutures and strips. - Hypothermia was actively prevented by using warming blankets, a warming mattress, and an intravenous fluid warmer. Post-operative: - Intraoperative and postoperative analgesia aimed to minimise narcotic use by using a multimodal combination of Paracetamol, Nefopam, Lidocaine, Ketamine, Tramadol, and Morphine - Prevention of postoperative nausea and vomiting consisted of intra-operative intravenous administration of Droperidol and/or Dexamethasone. - Chewing gum was suggested to the patient once awake and cooperative. - On return to the ambulatory surgical unit, patients received no intravenous perfusion; only oral analgesia was administered. - Within the following 2 h, patients were encouraged to walk and food was allowed.	Daily home visits by a nurse for the first 10 postoperative days	Pre-operative patient selection but discharge from PACU based on Aldrete score, discharge from hospital allowed based on Chung score [39] Patient selection criteria included the absence of serious or decompensated comorbidity, very good general condition, and full patient understanding of the procedure. Patients were discharged from the post-anaesthesia care unit as soon as the Aldrete score reached 9 Discharge was authorized if the patient met the exit criteria according to the Chung score [39].	Discharged to own home

*SDD* Same-day discharge, *POD* Post-operative day, *ERAS* Enhanced recovery after surgery

### Patient selection for SDD and criteria for early discharge

Bednarski et al. [23] randomised patients to the SDD arm. Brandt et al. [24] allowed patient opt-in for SDD. Most prospective studies [22, 25–27, 29, 31–33, 35] selected patients for SDD based on clinical and social characteristics. Dobradin et al. [28] and McKenna et al. [30] reported retrospective studies without elaboration of SDD selection criteria. For most studies, the final decision for early discharge was made by the surgeon, without a standard discharge checklist. However, McKenna et al. [30], and both studies by Gignoux et al. [22, 35] only discharged patients early if they met an objective set of discharge criteria.

### Study quality

Most studies had an overall low risk of bias based on the ROBINS-I tool (Supplementary Fig. 1) and were deemed to be methodologically robust. Three studies, by de Azevedo et al. [27], Popeskou et al. [31], and Tweed et al. [32], had moderate risks of bias, mainly due to missing data and selection of participants.

### Primary outcomes

All outcomes are summarised in Table 3.

**Table 3:** Outcomes of interest of studies included in the systematic review of SDD vs standard ERAS following major colorectal surgery

	Perioperative outcomes						
First author, Year	Bleeding	Operation time	Conversion	Bleeding	Pain	Infection	Anastomotic leaks
Bednarski et al. (2019) [23]	Not stated	Not stated	Not stated	Not stated	Mean Brief Pain Inventory (BPI) score at discharge P = 0.052 SDD: 3.23 (sd 1.86) Control: 1.8 (sd 1.63)	Not stated	SDD: 1
Brandt et al. (2013) [24]	Median P = 0.498 SDD: 20 (range 0–100) ml Control: 10 (range 0–1700) ml	Median P = 0.002 SDD: 120 (range 82–220) mins Control: 155 (range 85–350) mins	Not stated	Not stated	Not stated	Not stated	Not stated
de Azevedo et al. (2021) [27]	Not stated	Data only available for SDD patients, and only 236 patients Did not specify mean/median P = 0.021 70 min (55.0–100.0)	Not stated	Not stated	Not stated	1 that required readmission	3 that required readmission
Lee et al. (2022) [29]	Median estimated blood loss P = 0.089 SDD (out of the 48 patients): 5 ml (IQR 5–100) Control: 100 ml (IQR 28–200)	< 0.001 SDD (out of the 48 patients): 116 min (sd 56) Control: 177 min (sd 74)	Not stated	SDD (out of the 48 patients): 2 Control: 1	Not stated	Superficial/ deep SSI SDD (out of the 48 patients): 2 (4.2%) Control (out of 73 patients): 2 (2.7%)	Anastomotic leak/organ space SSI SDD (out of the 48 patients): 1 (2.1%) Control (out of 73 patients): 3 (4.1%)
McKenna et al. (2020) [30]	Not stated	Median P < 0.0001 Discharged POD 0–1: 95 min (IQR 66–141) Discharged POD 2: 143 min (IQR 106–185) Discharged POD 3–5: 159 min (IQR 119–209)	Not stated	Not stated	Not stated	Superficial SSI P < 0.01 Discharged POD 0–1: 16 (1.8%) Discharged POD 2: 108 (1.6%) Discharged POD 3–5: 663 (2.3%) Deep SSI P = 0.05 Discharged POD 0–1: 1 (0.1%) Discharged POD 2: 9 (0.1%) Discharged POD 3–5: 82 (0.3%) Sepsis or septic shock P = 0.06 discharged POD 0–1: 1 (0.1%) discharged POD 2: 50 (0.7%) discharged POD 3–5: 233 (0.8%)	P = 0.05 Discharged POD 0–1: 5 (0.6%) Discharged POD 2: 68 (1.0%) Discharged OD 3–5: 356 (1.2%)
Popeskou et al. (2022) [31]	Not stated	SDD: 150 min (range 125–180) Control:135 min (range 110–175)	Not stated	Not stated	Not stated	Not stated	Not stated
Levy et al. (2009) [33]	Not stated	P = 0.17 SDD: 73 min (range 50–110) Control: 88 min (range 50–160)	Not stated	Not stated	Mean pain scores at rest and with movement on the morning of discharge: At rest: 1.5 (range, 0–4) With movement: 3.3 (range,1–6)	Not stated	Not stated
Tweed et al. (2022) [32]	Not stated	Not stated	0	Rectal blood loss SDD (out of 41): 4 (7.3%) Control: 0	Not stated	SDD (out of 41): 1 UTI (7.7%) 1 pneumonia (7.7%) Control: 2 abscess (10%) 4 pneumonia (20%) 1 PID (5%) 1 infection e.c.i. (5%)	SDD (out of 41): 1 (7.7%) Control: 4 (20%)
Campbell et al. (2022) [25]	Not stated	Not stated	Not stated	Not stated	Not stated	0	0
Curfman et al. (2022) [26]	Not stated	Mean operative time by type of surgery (min) LAR (61): 163 R col (25): 161 Cecetomy (9): 53 Sigmoidectomy (6): 103 Parastomal hernia (5): 122 Transverse col (3): 135 Proctectomy (3): 216 L col (3): 135 Overall mean operative time: 149	0	Not stated	Not stated	0	0
Dobradin et al. (2013) [28]	Intraoperative estimated blood loss 74.3 ml	118 min (range 65–158)	Not stated	Not stated	Mean maximal pain score (on scale of 0 to 10) 1.9 (range 1–3)	Not stated	Not stated
Gignoux et al. (2015) [22]	Not stated	128 min (range 50–180)	Not stated	Not stated	Mean pain score via verbal descriptor scale (VDS) on Day 1: 1.6 Day 3: 2.7 Day 5: 1.55	0	0
Gignoux et al. (2019) [35]	Not stated	Not stated	Not stated	SDD (out of 157 patients): - Rectal bleeding: 2(1.3%)	SDD (out of 157 patients): - Mean maximum pain recorded on POD 1: 2.9 (sd 0.5) - Mean maximum pain recorded on POD 2: 2.7 (sd 0.5) - Mean maximum pain recorded on POD 3: 2.2 (sd 0.4)	SDD (out of 157 patients): - Wound abscess: 3 (1.9%) - Colitis: 1 (0.6%)	SDD (out of 157 patients): 3 (1.9%)

	Post-operative outcomes					
First author, Year	Mortality	Mean LOS	Readmission rate	Complication rate	Protocol Compliance	Patient satisfaction
Bednarski et al. (2019) [23]	Not stated	Mean initial LOS P = 0.002 SDD: 28.2 (sd 10.7) h Control: 53.8 (sd 17.8) h Mean total 30-day total LOS P = 0.041 SDD: 80.4 (sd 142.6) h Control: 53.8 (sd 17.8) h	SDD: 2 (1 anastomotic leak, 1 bowel obstruction from port-site hernia) Control: 0	P = 0.209 SDD: 5 (35.7%)complications, 3 patients with complications, 2 Clavien Dindo grade ≥ III 3 Clavien Dindo grade I-II (Both patients with grade 3 complications also had grade 2 complications) Control: 0 (0.0%)	Not stated	Patient satisfaction was assessed at POD 30 using a 20-question custom-designed survey No significant differences between the treatment arms for any of the questionnaire items. (P = 0⋅462)
Brandt et al. (2013) [24]	Not stated	Mean LOS not stated but controls were discharged between POD2-4	P = 0.369 Readmission to hospital: SDD: 0 Control: 9 (4%)	P = 0.103 Post-operative complications: SDD: 0 Control: 20 (10%)	Not stated	Not stated
de Azevedo et al. (2021) [27]	P = 0.056 SDD: 0 Control: 8 (1.9%)	Not stated	30-day readmission rate: SDD: 16 (6.8%) - 10 Gastroparesis/ileus - 3 Anastomotic leak - 1 Local site infetion - 1 Internal hernia - 1 Incisional hernia	Not stated	Not stated	Not stated
Lee et al. (2022) [29]	Not stated	Median P < 0.001 SDD (out of the 48 patients): 0 days (IQR 0–0) Control (out of 73 patients): 2 days (IQR 1–4)	30-day readmission rate: P = 0.681 SDD (out of the 48 patients): 3 (6%) - 1 Abdominal pain (colitis) - 1 Diarrhoea and infection (c. difficile) - 1 Wound infection (deep SSI) Control: 3 (4%)	30-day complication rate: P = 0.813 SDD (out of 48 patients): 8 (17%) Control: 11 (15%)	Not stated	Patient satisfaction was assessed using a questionnaire scored using 5-level Likert scales. Patients were also provided a space to comment on postoperative symptoms and difficulties Satisfaction scores were returned by 35 patients, all of whom had a successful SDD 80% of respondents did not feel like they needed to stay in hospital for their recovery, and 80% would still choose to go home on the day of surgery if they had surgery again. There were also 80% of respondents that reported high satisfaction with the app.
McKenna et al. (2020) [30]	Not stated	Not stated	Overall readmission: P = 0.04 Discharged POD 0–1: 43 (4.8%) Discharged POD 2: 349 (5.1%) Discharged POD 3–5: 1670 (5.8%)	Overall complication rate not stated The post discharge rate of anastomotic leak in the early discharge group was only 1%, and this was similar to the anastomotic leak rate in the intermediate (1%) and standard (1%) discharge groups. The rate of both ileus (early: 2%, intermediate: 2%, standard: 2%) were similar across the 3 discharge groups, as were other postoperative complications	Not stated	Not stated
Popeskou et al. (2022) [31]	30-day mortality SDD: 0 (0%) Control:2 (0.3%)	Not stated	30-day readmission rate: SDD: 4 (7.8%) - 1 SBIO from adhesions - 1 extraction site wound abscess - 1 nausea - 1 superficial abdominal pain Control: 72 (9.2%)	Not stated	100% adherence rate	Not stated
Levy et al. (2009) [33]	Not stated	Median post-operative stay P = 0.01 SDD: 0.95 days (IQR 0.95–0.96) Control: 3.2 days (IQR 2.4–3.9)	0	0	Not stated	All patients were satisfied with the service; all ten would request to follow the same pathway again if required, and all would recommend it to other patients
Tweed et al. (2022) [32]	30 day mortality SDD: 0% Control:0%	Median primary length of hospital stay P = 0.000 SDD (out of 41): LOS 1 day: 33 (80.5%) LOS 2 days: 6 (14.9%) LOS 3 days: 2 (4.9%) Control: LOS 1 day: 0 LOS 2 days: 9 (12%) LOS 3 days: 38 (50.7%) LOS > 3 days: 28 (37.3%)	30-day readmission rate: P = 0.051 SDD (out of 41 pts): 7 (17.1%) - 1 Anastomotic leak - 1 Trocal hernia - 2 Rectal blood loss - 1 Ileus - 1 Pneumonia - 1 Dizziness Control: 4 (5.3%) - 1 Anastomotic leak - 1 Ileus - 1 Biliary pancreatitis - 1 Abscess Patients discharged < 23 h (out of 33 patients): 6 (18.2%)	P = 0.565 SDD (out of 41 pts): 13 (31.7%) Control: 20 (26.7%) Patients discharged < 23 h (out of 33 patients): 9 (27.3%) Severe complications (Clavien Dindo grade IIIb-IV) SDD: 2 (4.9%) Control 6 (8.0%)	Not stated	Evaluation forms assessing patients’ satisfaction were returned by 39 patients (95%) The CHASE protocol was positively reviewed by the majority of patients. 87% of patients rated the program as an 8 or higher on a scale of 10.
Campbell et al. (2022) [25]	Not stated	Not stated	0	Overall complication rate not stated None suffered complications such as anastomotic leak, surgical site infection, or bowel obstruction or required admission to the hospital 1 patient was seen in the emergency department on POD 1 for nausea and vomiting and was managed as an outpatient. 1 patient required a fluid bolus in the clinic for high ileostomy output.	Not stated	Not stated
Curfman et al. (2022) [26]	0	Not stated	30-day readmission rate: 0% for all surgeries except 0.9% (n = 1) for sigmoidectomy due to post-op urinary retention	Causes of 30-day ED visit - Abdominal pain: 2 (1.7%) - Urinary retention: 1 (0.9%) - Leg pain: 1(0.9%) - Diarrhoea: 1(0.9%)	Not stated	Not stated
Dobradin et al. (2013) [28]	Not stated	21 h 47 min (range 18 h 31 min to 23 h 56 min)	Not stated	Not stated	Not stated	Not stated
Gignoux et al. (2015) [22]	0	10.85 h (range 10 h 15 min to 12 h)	0	Complications in 2 patients (none readmitted) - 1 Dysuria - 1 Incisional hematoma	Not stated	The results of the patient satisfaction questionnaire indicated an excellent appreciation for all patients; they would recommend the program to a close friend
Gignoux et al. (2019) [35]	Not stated	Mean LOS 10.4 h (sd 3.4)	9 readmissions (5.7%), of which 6 required re-operation: - 3 (1.9%)anastomotic leak - 2 (1.3%)SBIO - 1 (0.6%) omentum necrosis	30-day morbidity rate: 24.8% Clavien Dindo I: 28 (17.8%) Clavien Dindo II: 5 (3%) Clavien Dindo IIIA: 0 Clavien Dindo IIIB: 6 (4%)	Not stated	Not stated

Reported as n/%

*SDD* same-day discharge, *ERAS* enhanced recovery after surgery, *SSI* surgical site infection, *LOS* length of hospital stay, *SD* standard deviation

#### Readmission rates

The 30-day readmission rate in the intervention arm was low across all studies. Of the five single-arm studies, Campbell et al. [25], Dobradin et al. [28], and Gignoux et al. [22] reported zero readmissions within 30 days. Curfman et al. had a 0.9% (n = 1) readmission rate due to urinary retention [26], while Gignoux et al. had a 5.7% (9/157) readmission rate [35]. Of these nine patients, three had anastomotic leaks, two had small bowel intestinal obstruction and one had omental necrosis [35].

SDD protocols did not lead to increased 30-day readmission rates compared to standard ERAS. Six studies, by Bednarski et al. [23], Brandt et al. [24], Lee et al. [29], Popeskou et al. [31], Tweed et al. [32], and Levy et al. [33], reported no significant differences in 30-day readmission rates between both groups. The remaining two studies did not publish readmission rate data for the control group but reported a 30-day readmission rate of 6.8% and 0% in the SDD group [27, 33].

#### Length of hospital stay (LOS)

Of the five single-arm studies, three studies, by Gignoux et al. [22], Dobradin et al. [28] and Gignoux et al. [35] reported mean LOS, which ranged from 10 h 24 min [35] to 21 h 47 min [28]. Four comparative studies, by Bednarski et al. [23], Lee et al. [29], Levy et al. [33], Tweed et al. [32] reported mean or median LOS, showing significantly reduced LOS in the SDD group compared to the control group. Bednarski et al. demonstrated that SDD reduced the LOS by 25.6 h as compared to standard ERAS (28.2 h vs 53.8 h, P = 0.002) [23]. Lee et al. reported that the median LOS in the SDD arm was reduced by 2 days as compared to standard ERAS (0 days vs 2 days, P < 0.001) [29]. Levy et al. demonstrated a 2.25 day reduction in median post-operative LOS in SDD versus conventional ERAS arm (0.95 days vs 3.2 days, P = 0.01) [33]. Finally, Tweed et al. showed that majority of their SDD cohort had a median primary LOS of 1 day (80.5%), compared with their ERAS cohort with majority of patients having a median primary LOS of 3 days (50.7%, P < 0.001) [32].

### Secondary outcomes

#### Operative time

Two studies reported significantly quicker operative times with SDD. Brandt et al. reported a 35 min reduction in median operative time in the SDD arm compared to standard ERAS (120 min vs 155 min, P = 0.002), for mostly sigmoid resections and right hemicolectomies [24]. Lee et al. demonstrated a 61 min reduction in mean operative time with SDD versus conventional ERAS (116 min vs 177 min, P < 0.001) [29]. Levy et al. also demonstrated quicker median operative times in the SDD group compared to control (73 min vs 88 min, P = 0.17) [33], although this was not statistically significant. Mckenna et al. showed that median LOS significantly increased as the operative duration increased [30]. Patients discharged POD 0 to 1 had a median operative time of 95 min (IQR 66–141 min), while patients discharged on POD 2 and 3 had significantly longer median operative times of 143 min (IQR 106—185 min) and 159 min (IQR 119—209 min) respectively (P < 0.0001) [30]. This reflects the longer recovery period required for more complex surgery.

#### Intraoperative blood loss

SDD protocols had similar intraoperative blood loss compared with standard ERAS, demonstrated by two studies; Lee et al. (Median blood loss: 20 ml vs 10 ml, P = 0.498) [29], and Brandt et al. (Median blood loss: 5 ml vs 100 ml, P = 0.089) [24].

#### Conversion

None of the studies reported conversions from minimally invasive to open surgery.

#### Postoperative morbidity

Six [23, 24, 29, 30, 32, 33] of the eight comparative studies reported complication rates following both SDD and standard ERAS, while the remaining two [27, 31] did not report complication rates at all. All six studies found similar morbidity rates between the SDD and standard ERAS. Notably, McKenna et al. reported significantly lower superficial infection rates (1.8% vs 2.3%, P < 0.01) in the SDD cohort as compared to those discharged from postoperative day 2 to 5 [30]. Deep surgical site infection rates (0.1% vs 0.3%, P = 0.05) and anastomotic leak rates (0.6% vs 1.2%, P = 0.05) were also lower in the SDD group, although these differences did not reach statistical significance [30].

All five single-arm studies evaluated postoperative morbidity [22, 25, 26, 28, 35]. Campbell et al. reported one patient with nausea and vomiting and one with high ileostomy output [25]. Curfman et al. reported 30-day emergency department visits, including two patients with abdominal pain, and one with urinary retention, one with leg pain and another with diarrhoea [26]. Gignoux’s 2015 study reported one patient with dysuria and one with an incisional hematoma [22]. The prospective study by Gignoux et al. in 2019 reported an overall 30-day morbidity rate of 24.8% with SDD, of which 72% were Clavien-Dindo grade I [35]. Dobradin et al. reported zero complications with SDD [28].

Four [22, 25, 26, 28] of the five single-arm studies reported no high-grade complications requiring reoperation. Gignoux’s 2019 study, comprising 157 patients, reported two patients with rectal bleeding, three with wound abscesses, one with colitis, three with anastomotic leak, two with small bowel intestinal obstruction, and one patient with omental necrosis [35].

#### Postoperative pain

One comparative study measured postoperative pain using the Brief Pain Inventory (BPI) score [23], with a higher mean discharge pain score in the SDD compared to the control arm (3.23 vs 1.80, P = 0.052), although scores were low in both arms. Furthermore, the authors measured discharge pain scores 1 day earlier in the SDD arm as compared to the control arm. BPI scores were similar post-discharge and at postoperative day 30. Three single-arm studies measured pain using the visual analogue scale rating from 0 to 10 [22, 28, 35]. Dobradin et al. reported a mean postoperative pain score of 1.9 with SDD [28]. In 2015, Gignoux et al. reported mean pain scores for SDD patients on postoperative days 1 (pain score 1.6), 3 (pain score 2.7) and 5 (pain score 1.5), demonstrating low scores throughout [22]. In 2019, Gignoux et al. again reported low mean pain scores amongst patients on SDD protocols across postoperative days 1 (pain score 2.9), 2 (pain score 2.7), and 3 (pain score 2.2) [35].

#### Postoperative nausea and vomiting (PONV)

PONV rates were generally low amongst studies reporting this outcome. Tweed et al. reported one case of prolonged LOS due to nausea [32]. Campbell et al. had one patient return to the emergency department on postoperative day 1 for PONV [25]. Gignoux et al. (2019) reported two readmissions amongst SDD patients for PONV [35].

#### Patient satisfaction

Five studies evaluated patient satisfaction [22, 29, 32, 33, 35]. Of these, only one study compared patient satisfaction between SDD and standard ERAS and found no significant differences between the treatment arms for any of the questionnaire items [22]. Nearly all respondents felt that they did not have to be kept in the hospital for a longer period to recover from surgery. In the other four studies, patient satisfaction was only assessed in the SDD arm [22, 29, 32, 33]. All four studies reported that most respondents were highly satisfied with the SDD protocol. Levy et al. [33] and Gignoux et al. [35] reported that most SDD patients would recommend this programme to others.

#### Protocol compliance

Popeskou et al. reported a 100% adherence rate to the ERAS protocol [31]. No other studies evaluated patient or clinician compliance to individual components of the ERAS or SDD protocols.

## Discussion

This is the first systematic review investigating the safety and efficacy of same-day discharge (SDD) protocols, or “hyper-ERAS”, after colorectal surgery, showing a significant reduction in LOS, low risks of readmissions, and comparable risks of morbidity. The benefits of a reduced LOS are numerous and include a reduction in nosocomial infections [36], thromboembolic events [37], healthcare costs [38], and improvement in morbidity and mortality rates while enhancing quality of life and patient satisfaction [37].

The safety and acceptability of SDD protocols depends on a multitude of factors, including patient factors (physiological fitness or presence of comorbidities, disease status, compliance to the pathway), surgical factors (type or complexity of surgery, anaesthetic protocols), and the extent of postoperative assessment or care (teleconsultations, home visitations, etc.). Each of these are key tenets of the reviewed studies and should be considered prior to initial implementation of SDD programmes.

### Patient factors

Patients selected to undergo SDD were invariably fitter individuals without serious health problems or poorly controlled chronic medical conditions, which would otherwise have heightened perioperative risks. The high rate of successful SDD amongst patients selected for SDD programmes amongst the reviewed studies reflects the appropriateness of patient selection. Cognitive impairment, high ASA scores, or the presence of significant comorbidities were frequently used exclusion criteria for SDD. Others excluded patients with a history of major or complex abdominal surgeries, or those with prior severe postoperative nausea or vomiting. Patients with poor health literacy, without suitable caregivers, should also be excluded from SDD.

For a more objective evaluation for fitness for discharge, several studies [22, 30, 35] used the Chung criteria [39], consisting of five variables including vital signs (temperature, pulse, respiration), ambulatory status, nausea and vomiting, pain, and surgical bleeding. A cut-off score of less than 9 out of 10 was used for patient exclusion from SDD. Gignoux et al. employed a 2-stage discharge process [22, 35]. First, the Modified Aldrete score [40] was used to discharge patients from the post-anaesthesia care unit. This score consists of aspects of motor activity, breathing, blood pressure, level of consciousness and oxygen saturation. Patients with a score above 9 were transferred from PACU to the ambulatory surgical unit. Patients were subsequently discharged from hospital in the evening if they met the Chung discharge criteria.

### Surgical and anaesthetic factors

Patients scheduled for more complex surgeries e.g., multivisceral resections, low rectal resections, resections requiring bowel diversion with ostomy, etc., should not be considered for SDD. Moreover, prolonged surgery, major intraoperative complications or unanticipated difficulties can compromise postoperative recovery and warrant exclusion from SDD protocols at the discretion of the operating surgeon or anaesthetist.

Laparoscopy and other minimally invasive surgery (MIS) options reduce operative trauma to the abdominal wall, reducing postoperative pain, analgesia use [41], improving LOS, as well as decreasing rates of readmission and reoperation [42]. It is unsurprising that MIS techniques have become the cornerstone of colorectal ERAS and SDD protocols. All patients who underwent SDD in this review had MIS colorectal surgery. Several operative variations can further improve patient recovery by minimising operative trauma and pain, including intracorporeal versus extracorporeal anastomosis for laparoscopic right hemicolectomy [43]. Pfannenstiel specimen extraction versus midline specimen extraction [44], or natural orifice specimen extraction versus conventional transabdominal specimen extraction [45, 46]. Individual surgeon expertise and experience are important factors [47, 48], as well as proficiency with the surgical platforms used, including conventional laparoscopy, robotic surgery, or transanal approaches.

The type of anaesthesia and postoperative analgesia administered also influences recovery. Opioid-sparing modalities can reduce postoperative ileus, nausea and urinary retention [9, 10], all of which can prolong LOS. Transversus abdominis plane (TAP) block has been shown to provide effective pain relief resulting in a significant reduction in opioid use following colorectal surgery [49, 50]. This method was employed by five of the reviewed studies [25–27, 29, 35]. In a recently reported randomised controlled trial, bilateral erector spinae plane (ESP) block was also demonstrated to reduce pain scores and opioid requirements for 24 h following laparoscopic colorectal surgery [51]. While this method was not employed by any of the reviewed SDD studies, it may be a viable alternative to TAP block for SDD pathways.

The 2022 American ERAS guidelines for colorectal surgery strongly recommend pre-emptive, multimodal antiemetic prophylaxis to reduce PONV [10]. Apart from utilisation of opioid-sparing analgesia and reducing operative time, decreasing inhalational anaesthesia can also reduce the risk of PONV [10]. Propofol-based total intravenous anaesthesia (TIVA) has been shown to lower the risk of PONV, reducing post-extubation pain scores and time spent in the post-anaesthesia care unit, while improving patient satisfaction scores, compared to inhalational anaesthesia [52]. Four of the reviewed studies reported PONV amongst SDD patients [25, 32, 33, 35]. Two studies reported administration of intraoperative droperidol and dexamethasone to reduce the risk of PONV [22, 35]. Tweed et al. initially administered a combination of spinal anaesthesia with bupivacaine-glucose and morphine intrathecally prior to general anaesthesia [32]. However, after the first nine patients, this was switched to 60–80 mg intrathecal prilocaine in combination with TIVA, because of an observed increase in incidence of PONV and urinary retention [32].

### Postoperative home monitoring

Home assessment of patients for post-SDD is essential for recognition of serious morbidity which may require urgent interventions. Adequate counselling should be provided to patients or caregivers, particularly advice concerning the early symptoms of potential complications. Additionally, a convenient channel for communication and/or evaluation between patients, caregivers, and trained healthcare professionals during the early post-discharge period allows for reassurance for minor postoperative issues, including expected gastrointestinal symptoms, or outpatient treatment for minor complications instead of hospital readmission. The postoperative complication rate and distribution of Clavien-Dindo grade of severity were similar between SDD and standard ERAS arms in the comparative study by Tweed et al. [32]. However, the readmission rate in the SDD group was more than threefold that of the control group (17.1% vs 5.3%, P = 0.051) [32], with outpatient treatment potentially possible in more than half of SDD readmissions.

Availability of and compliance to remote health assessments, as well as familiarity with digital platforms, are important aspects of SDD pathways. Five studies monitored patients via telephone calls with early clinic follow-up appointments [25–27, 32, 33]. Bednarski et al. described a “telerecovery” method, where patients underwent videoconferencing with their physicians following discharge [23]. The study protocol also allowed for outpatient intravenous fluid hydration at an ambulatory infusion centre when the patient was identified to have inadequate oral intake or assessed to be at high risk of dehydration [23]. Lee et al. piloted a mobile application that enabled direct communication between patients and physicians, with the added function of daily patient-reported health questionnaire administration [29].

Assessment of individual comfort level with remote postoperative monitoring, in lieu of physical ward rounds, is required. This will likely vary across geographical locations and healthcare settings, and is further dependent on several factors including patient or caregiver tech-savviness or proximity of their residence to the hospital. Appropriate preoperative counselling and reassurance will likely increase patient confidence with remote monitoring modalities. Two studies instituted early healthcare worker home visitation following discharge [22, 35]. Gignoux et.al. instituted a post-discharge protocol consisting of daily home visits by a nurse for the first 10 postoperative days [35]. These resource-intensive initiatives may not be cost-effective in the long-term and modifications can be made once the feasibility of SDD pathways are demonstrated.

### Limitations

The review is limited by the paucity of randomised trials comparing SDD with traditional ERAS. Moreover, there was marked heterogeneity in study designs, with significant variability in inclusion criteria, types of surgery, methods of anaesthesia, and discharge criteria, or other components of SDD protocols. The overall compliance rates to SDD or conventional ERAS protocols was also mostly unreported. In addition, not all studies reported the reasons for failure of SDD amongst patients included within SDD pathways. This may be relevant as discharge delays can often be due to social or non-medical reasons, clinician or patient preferences, as well as private insurance requirements [53].

The strict inclusion criteria for SDD limits its generalisability. Differences in patient demographic, surgical, or disease characteristics can result in confounding. SDD patients were significantly younger than their conventional ERAS counterparts in many studies [24, 27, 32, 33]. Most studies did not adjust for confounders during their analysis. Publication bias may ensue when studies with favourable outcomes are preferred over those with negative or inconclusive findings [54]. Lastly, selective reporting of outcomes, including omitting unfavourable or statistically insignificant outcomes, or publishing only a subset of analysed data, can undermine the validity of results.

## Conclusion

SDD protocols appear to be safe and feasible in a select group of patients undergoing major colorectal operations, resulting in reduced LOS with no increased risk of 30-day readmissions and postoperative morbidity compared to traditional ERAS. Randomised controlled trials are necessary to further substantiate these findings.

### Supplementary Information

Below is the link to the electronic supplementary material.

Supplementary file1 (DOCX 444 KB)

## Data Availability

Not applicable.

## Declarations

This manuscript has not been presented at a podium or poster meeting.
