# Supplementary figures and images for: Same-day discharge (SDD) vs standard enhanced recovery after surgery (ERAS) protocols for major colorectal surgery: a systematic review
Source: Int J Colorectal Dis. 2023 May 1;38(1):110. doi: 10.1007/s00384-023-04408-7 (PMC10149457; doi:10.1007/s00384-023-04408-7)

**Supplementary Figure 1: ROBINS-I tool summary**

**
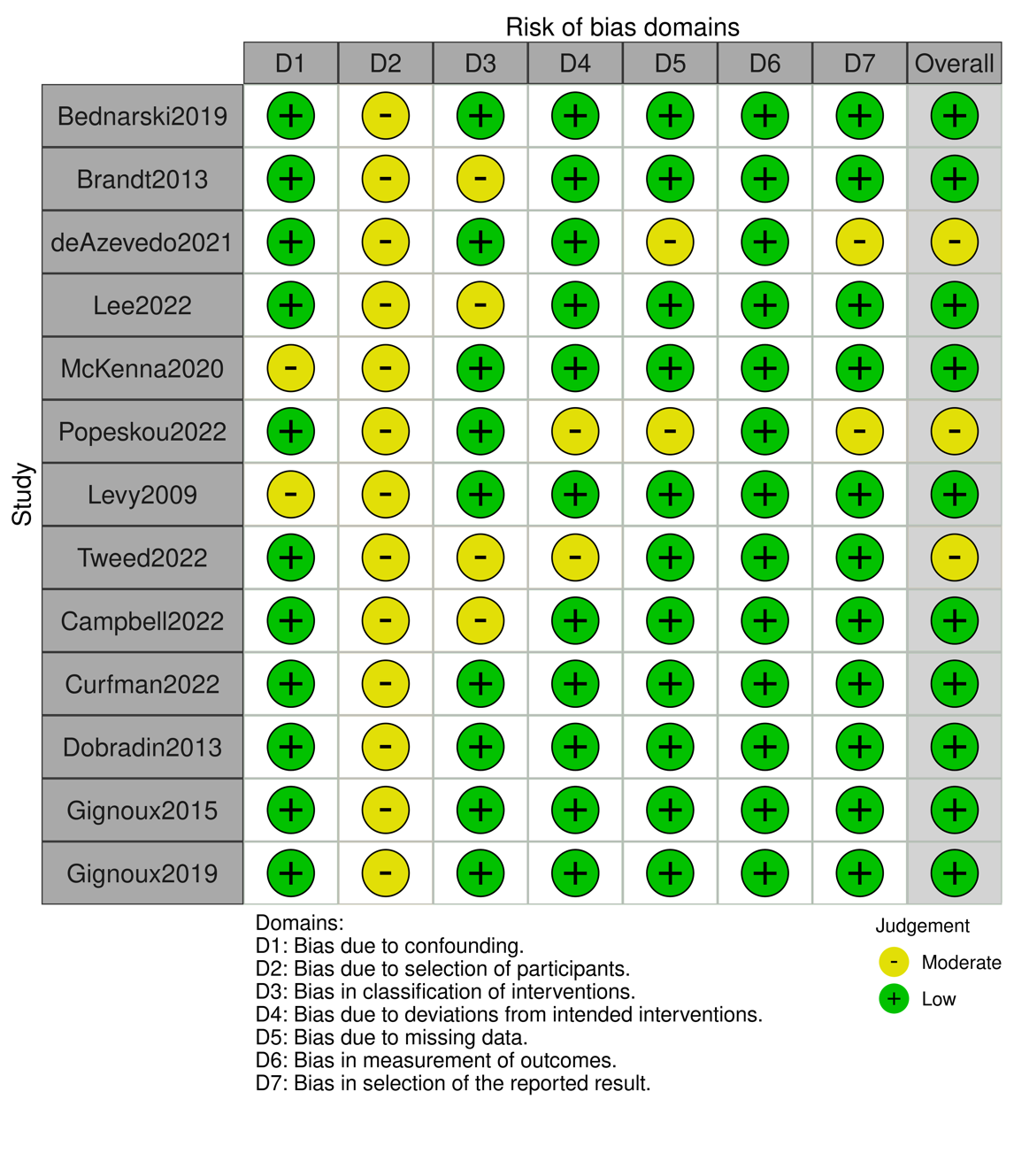
**

Supplement: Supplementary file 1 — Supplementary file1 (DOCX 444 KB) [file 384_2023_4408_MOESM1_ESM.docx]
